# Editorial: Psycho-criminology and forensic psychiatry: the intersections of mental health and the law

**DOI:** 10.3389/fpsyt.2023.1237466

**Published:** 2023-06-27

**Authors:** Heng Choon (Oliver) Chan

**Affiliations:** Department of Social Policy, Sociology, and Criminology, School of Social Policy, University of Birmingham, Birmingham, United Kingdom

**Keywords:** psycho-criminology, forensic psychiatry, mental health, law, psychological criminology

Broadly speaking, forensic psychiatry is the scientific study of the relationship between mental disorders and criminal behavior in a forensic context, and the application of psychiatric knowledge to crime, criminal and civil law, and the impact of law on human behavior. Psycho-criminology (or simply as psycho-criminology) is generally concerned with the use of psychological knowledge and skills (i.e., the study of human behavior and mental processes) in explaining, describing, and potentially preventing deviant, delinquent, and offending behavior (1–6). More specifically, Bartol (7) regarded psycho-criminology as the scientific study of criminal behavior, especially on how the behavior is learned, evoked, sustained, and changed consequent to the influences of human personality, social situations, and environmental conditions. Wortley (8) asserts that the subdiscipline of psycho-criminology simply addresses the overarching question of “What is it about the individuals and their experiences that cause them to commit crime and/or to become criminal?” (p. 1).

The emphasis of this Research Topic (RT) focuses on the application of psycho-criminological approaches and constructs to criminal behavior and mental health in forensic settings. There are six articles included in this RT, which explored various features of crime and delinquent/criminal behavior through the application of psychological and criminological concepts and theories. Contributions to this RT are geographically diverse, with topics covering Africa (Nigeria), Asia (Hong Kong and mainland China), Europe (the UK), and the Middle East (Iran). More importantly, the collection of these articles addresses varied features of deviant and criminal behavior (e.g., risky sexual behaviors, paraphilic interests, and psychopathic traits), delinquency and crime (e.g., sexual offending and corruption), and in the forensic settings (e.g., prison and criminal court).

The first article in this RT is an empirical study of family functioning and adolescent delinquency in a large sample of mainland Chinese adolescents by Shek et al.. This article examines the relationship between family functioning and adolescent delinquency, and the mediating role of positive youth development (PYD) attributes on this relationship. Analyzing a two-wave short-term longitudinal data (6 months apart) collected from 4,922 mainland Chinese adolescents, findings are that family functioning at the initial period (Wave 1) were negatively associated with the level of and change in delinquent behavior at the subsequent period (Wave 2), and PYD attributes (Wave 2) are found to mediate this relationship. Also, the general strain theory was tested by Wang et al. by surveying 687 mainland Chinese inmates recruited from 60 prisons, who were former public officials convicted of corrupt behaviors. They aim to explore the potential mediating effect of strain on the public officials' corrupt behaviors. Their findings indicate that clerks (a higher rank) and non-clerks (a lower rank) tended to have different experiences of status-related strain and personal financial strain, but it is the work-related strain that significantly distinguishes them. Compared to non-clerks, the work-related strain has more significant impact on clerks.

Examining the dynamics of sexual offending among youth in Hong Kong, Chan recruited a nonclinical sample of 863 young people aged 17–20 years to examine the psychosocial risk factors of low self-control, risky sexual behavior (RSB), and paraphilic interests. Men are found to have significantly higher levels of threat of sexual assault and of general as well as of 12 subtypes of paraphilic interests than women, while women have a higher level of paraphilic interest on transvestic fetishism than men. Low self-control and more RSB and paraphilic interests are generally significant predictors of sexual assault threat issuance and perpetration of both penetrative and nonpenetrative sexual assault. Next, Ghazanfari et al. recruited a randomly selected sample of 259 male prisoners in Iran to examine their psychological wellbeing, family cohesion, and purposeful life. They found that the inmates' psychological wellbeing is significantly positively associated with their family cohesion, but both psychological wellbeing and family cohesion are not significantly correlated with their sense of a purposeful life.

In the UK, Lilley et al. sampled a group of 108 jury-eligible university students to partake in one of nine identical 12-person mock trial simulations (pre- and post-trial) depicting a videotaped stage-setting of an intimate partner rape trial. Investigating the role of jurors' traits of psychopathology, crime-specific attitudes, sexual victimization experience, and demographic characteristics, the authors found that rape myth beliefs and juror ethnicity are positively associated with verdict decisions both pre- and post-deliberation. Besides, a diminished affective responsiveness (empathy) and experience of sexual victimization significantly predicted guilty verdict selections by the participants in the post-trial deliberation. Similarly, from a legal perspective, Ogunwale et al. critically examined the insanity plea in the Nigerian legal system. Adopting reform-oriented and fundamental legal research and psycho-legal formulated illustrative cases, the authors contend that there is a misalignment between the subjective experience of the mentally ill defendant and the objective judicial unawareness of the defendant's psychiatric conditions in some cases in Nigeria. As a result, a potential miscarriage of justice can occur. Hence, the authors argue for a reform based on current multidisciplinary knowledge whereby mental health professionals should be involved in all insanity plea cases.

Based on studies conducted in different countries, this RT collectively demonstrate the significance of applying psycho-criminological knowledge to advance our understanding of the fundamental mechanisms (i.e., personal, social, and environmental influences) related to offending behavior in the intersections of mental health and the law, especially in the forensic setting. In this emerging subdiscipline of psycho-criminology, it is utterly imperative to continue moving forward with more international research to advance our knowledge and contribute to the repertoire of literature, which can have implications for research, practice, and policy development or refinement. Importantly, given the geographically diversified studies in this RT, it is noteworthy to acknowledge the importance of cultural influences wherein culturally adapted psychiatric and psychological interventions should not be neglected (3).

## Author contributions

The author confirms being the sole contributor of this work and has approved it for publication.
